# A controlled trial of a dissonance-based eating disorders prevention program with Brazilian girls

**DOI:** 10.1186/s41155-019-0126-3

**Published:** 2019-06-17

**Authors:** Ana Carolina Soares Amaral, Eric Stice, Maria Elisa Caputo Ferreira

**Affiliations:** 1 Federal Institute of Education, Science and Technology of Southern of Minas Gerais, 204 Monsenhor José Augusto, São José, Barbacena, 36205-018 Brazil; 2 Oregon Research Institute, 1776 Millrace Drive, Eugene, OR 97403 USA; 3 Federal University of Juiz de Fora, Campus Martelos, Juiz de Fora, 36036-330 Brazil

**Keywords:** Eating disorder, Prevention, Dissonance, Body dissatisfaction

## Abstract

**Background:**

Given that most young women with eating disorders do not receive treatment, implementing effective prevention programs is a public health priority. The Body Project is a group-based eating disorder prevention program with evidence of both efficacy and effectiveness. This trial evaluated the efficacy of this prevention program with Brazilian girls, as no published study has tested whether this intervention is culturally sensitive and efficacious with Latin-American adolescents.

**Methods:**

Female students were allocated to a dissonance-based intervention (*n* = 40) or assessment-only (*n* = 22) condition. The intervention was a dissonance-based program, consisted of four group sessions aimed to reduce thin-ideal internalization. The sessions included verbal, written, and behavioral exercises. The intervention group was evaluated at pretest and posttest; assessment-only controls completed measures at parallel times.

**Results:**

Compared to assessment-only controls, intervention participants showed a significantly greater reduction in body dissatisfaction, sociocultural influence of the media, depressive symptoms, negative affect, as well as significantly greater increases in body appreciation. There were no significant effects for disordered eating attitudes and eating disorder symptoms.

**Conclusions:**

These results suggest that this dissonance-based eating disorder prevention program was culturally sensitive, or at least culturally adaptive, and efficacious with Brazilian female adolescents. Indeed, the average effect size was slightly larger than has been observed in the large efficacy trial of this prevention program and in recent meta-analytic reviews.

**Trial registration:**

RBR-7prdf2. Registered 13 August 2018 (retrospectively registered).

## Background

Eating disorders (ED) affect 15% of females and they are marked by chronicity, relapse, distress, functional impairment, and increased risk for future obesity, depression, suicide attempts, and mortality (Allen, Byrne, Oddy, & Crosby, 2013). Although there are no data about the prevalence of ED in Brazil, it has been established that its incidence has increased in recent years (Nunes, 2006). Researchers have argued that this increase could be because of greater understanding of this subject and the more accurate diagnosis of these disorders (Nunes, 2006; Prisco, Araujo, Almeida, & Santos, 2013). According to Smink, van Hoeken, and Hoek (2012), there has been an increase in the high risk-group of 15–19-year-old girls, who meet diagnostic criteria for subclinical conditions of ED.

As 80–90% of those with eating disorders do not receive treatment (Swanson, Crow, Le Grange, Swendsen, & Merikangas, 2011), a public health priority is to broadly implement effective eating disorder prevention programs focused in eating disorders risk factors, such as body dissatisfaction.

Many studies have revealed elevated body dissatisfaction among Brazilian female adolescents (e.g., Alves, Vasconcelos, Calvo, & Neves, 2008; Laus, Miranda, Almeida, Costa, & Ferreira, 2012; Martins & Petroski, 2015; Scherer, Martins, Pelegrini, Matheus, & Petroski, 2010; Vale, Kerr, & Bosi, 2011). Amaral and Ferreira (2017), in their 1-year follow-up, concluded that the media influence was the most powerful predictor of body dissatisfaction among Brazilian girls, which in turn, predicted risky eating attitudes. Similarly, other studies conducted in Brazil have found that media contributes to body image disturbances, particularly among adolescent girls and young women (Alvarenga, Dunker, Philippi, & Scagliusi, 2010; Fortes, Amaral, Almeida, & Ferreira, 2013).

One eating disorder prevention program with a broad evidence base is the Body Project (BP; Stice, Mazotti, Weibel, & Agras, 2000). This intervention is based on cognitive-dissonance theory, proposed by Festinger (1957), that suggest that people are motivated to maintain consistency between their behaviors and attitudes, and that when an individual engages in a behavior that is inconsistent with an attitude, they experience psychological discomfort that causes them to align their attitudes with their behavior. In this group-based eating disorder prevention program, adolescent girls voluntarily critique the thin beauty ideal in verbal, written, and behavioral exercises, which theoretically generates cognitive dissonance that prompts them to reduce their subscription to this unrealistic ideal because people are motivated to align their attitudes with their publically displayed behaviors.

BP is a four-session intervention wherein participants verbally generate costs associated with pursuing the thin ideal in response to Socratic questions, complete role-plays in which they talk facilitators out of pursuing this ideal, write a letter to a younger self on how to avoid body image concerns, and engage in acts of body activism that challenge this ideal (intervention script can be found at www.bodyprojectsupport.org). This intervention is based on The Dual Pathway Model that hypothesizes that thin-ideal internalization increases risk for body dissatisfaction, which in turn increases risk for subsequent dieting and negative affect, which increases risk for ED onset (Stice, 2001). Stice, Marti, Rohde, and Shaw (2011) confirmed that a decrease in thin-ideal internalization mediated the effects of the BP on body dissatisfaction, and further than a reduction of body dissatisfaction mediated the decline in eating disorders symptoms.

The BP is one of the few prevention programs to significantly decrease onset of eating disorders over follow-up in multiple trials, outperform active alternative prevention programs, and to produce effects in trials conducted by independent research teams in North America and Europe (e.g., Becker, Smith, & Ciao, 2005; Halliwell, Jarman, McNamara, Risdon, & Jankowski, 2015; Matusek, Wendt, & Wiseman, 2004; Serdar et al., 2014; Stice, Marti, Spoor, Presnell, & Shaw, 2008; Stice, Rohde, Shaw, & Gau, 2011). These trials have demonstrated that this cognitive-dissonance intervention has produced greater reductions in eating disorder risk factors (such as thin-ideal internalization, body dissatisfaction, dieting, negative affect) and eating disorder symptoms in adolescent girls and young women with body image concerns relative to assessment-only control conditions, and often relative to alternative interventions.

The meta-analytic review conducted by Le, Barendregt, Hay, and Mihalopoulos (2017) noted out that cognitive-dissonance interventions were effective in reducing risk factors (e.g., body dissatisfaction, thin-ideal internalization, negative affect, and dieting) and symptoms of eating disorders for late adolescents and young women. Moreover, the BP has produced effects when implemented selectively with women who have body image concerns, as well as when implemented universally to women and adolescents who were not screened for body image concerns or when implemented in an indicated fashion with women who have subclinical eating pathology (Stice, Shaw, & Marti, 2007).

It is essential to evaluate the efficacy of eating disorder prevention among different ethnic groups, in order to determine whether prevention programs need to be modified to fit the particular needs of different groups. Although there is some evidence that the BP is similarly effective for Asian American, African American, Hispanic, and European American females (Rodriguez, Marchand, Ng, & Stice, 2008; Stice, Marti, & Cheng, 2014), no randomized trials have evaluated the efficacy of the BP among young women from other cultures. Thus, the objective of the present study was to evaluate the efficacy of the BP for Latin-American girls in Brazil. We hypothesize that the BP will produce significantly greater reductions in body-image dissatisfaction, sociocultural influences by the media, disordered eating attitudes and behaviors, eating disorders symptoms, negative affect, and increase body appreciation in the intervention group than changes observed in assessment-only controls.

## Methods

### Participants and procedure

Initially, the sample size was calculated using as parameters the average effect size of the larger efficacy trial of BP from the comparison among intervention and assessment-only conditions (*d* = 0.56; Stice, Shaw, Burton, & Wade, 2006), the expected power (0.80), the significance level (*p* < .05), and the statistical test (mixed repeated measures ANOVA). The minimal sample size indicated to a representative sample was 80 participants.

Recruitment was conducted via flyers and was directed to adolescent girls from the technical education integrated to the high school of the “Instituto Federal do Sudeste de Minas Gerais”. This report is about girls recruited between August 2015 and March 2017. Girls with body image concerns were included in this study (accessed by the direct question “Do you have body image concerns?”). They also should not met criteria for eating disorders (accessed by the direct question “Have you ever been diagnosed with some kind of eating disorder (e.g., anorexia and bulimia)?”). Therefore, this represents a selective prevention program.

One hundred forty one adolescent females (*M* age = 16.25, *SD* = 1.4) accepted the invitation to enroll the trial. Girls that voluntarily accept take part in the study were randomized to the intervention or assessment-only condition through the website www.randomization.com. In total, 79 participants were assigned to the BP condition and 62 participants were assigned to an assessment-only condition.

**Fig. 1:**
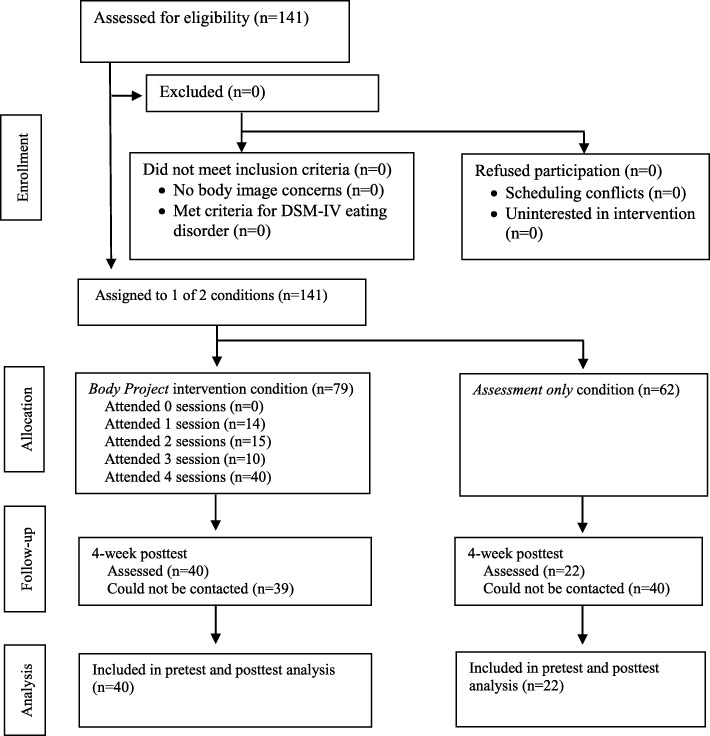
Participant flow throughout study

The final sample in the posttest was composed by 40 participants in the intervention group, that completed the four-session protocol, and 22 assessment-only controls, who answered the questionnaire in both pre- and posttest (see Fig. Fig. 1 for a participant flowchart).

#### Intervention

The BP consisted of four weekly 1-h group and each intervention group included five to eight participants. Facilitators delivered the intervention using a scripted manual. Participants completed assessments at pretest and posttest and the assessment-only controls completed measures at parallel times. All the measures were self-reported with no limit of time to answer the questionnaires.

The scripted intervention manual was translated into Portuguese, aiming to be as faithful as possible to the original (see Table 1 for the script of each session and visit www.bodyprojectsupport.org for the Portuguese translation of the intervention script). The sessions took place at the school’s premises, after school hours, according the availability of the participants. The intervention was implemented by researchers trained in the intervention protocol. As done in other trials, we underscored the voluntary nature of participation and increased accountability (e.g., by videotaping sessions) to increase dissonance induction. This is justified because the voluntary engagement in counter-attitudinal activities, such as exploring costs of pursuing the thin ideal, produces greater attitudinal change (Green, Scott, Diyankova, Gasser, & Pederson, 2005; Stice, Shaw, Becker, & Rohde, 2008).

**Table 1:** Description of the Body Project intervention

Session	Description
1	In session 1, participants collectively define the thin ideal, discuss costs of pursuing this ideal, and are assigned home exercises (e.g., write an essay about the costs associated with pursuing the thin ideal).
2	In session 2, participants discuss each home exercise, dissuade facilitators from pursuing the thin ideal in role-plays, and are assigned more exercises (e.g., generate a top 10 list of things young women can do to challenge the thin ideal).
3	In session 3, participants discuss home exercises, conduct role-plays challenging thin-ideal statements, discuss personal body image concerns, and are assigned home exercises (e.g., engage in a behavior that challenges their body image concerns).
4	In session 4, participants discuss home exercises, plan for future pressures to be thin, discuss perceived benefits of the group, and are assigned exit home exercises (e.g., engage in a group body activism).

Procedures were approved by the Research Ethics Committee on Human Beings of “Universidade Federal de Juiz de Fora” (n 228.386).

### Measures

#### Primary outcomes

##### Body dissatisfaction

Body weight and shape concerns were assessed with the 34-item Body Shape Questionnaire (BSQ). Scores ranged from 34 to 204, wherein the higher the score, the greater the body dissatisfaction. This scale has shown internal consistency (α = .96) and test-retest reliability (*r* = .91) for Brazilian adolescents (Conti, Cordás, & Latorre, 2009). According to the final scores, the girls were classified as having no dissatisfaction (scores of less than 80), with slight dissatisfaction (scores between 80 and 110), moderate dissatisfaction (scores between 111 and 140), or serious dissatisfaction (scores higher than 140). The internal consistency of BSQ for the present study sample, as evaluated using Cronbach’s alpha, was .95 at pretest and .93 at posttest.

##### Sociocultural influences

The 30-item Sociocultural Attitudes Towards Appearance Questionnaire-3 (SATAQ-3) assessed the influence of the media on body image, including thin-ideal internalization, pressures to be thin, and the media as source of information about appearance. The final score was calculated by the sum of the responses, ranged from 30 to 150, and the score proportionally represented the influence of sociocultural aspects on body image. This scale has shown internal consistency (α > .91), 2-week test-retest reliability (*r =* .86), and factorial structure among Brazilian adolescents (Amaral, Conti, Filgueiras, & Ferreira, 2015). Cronbach’s alpha in the present study sample was .94 at pretest and .90 at posttest.

##### Disordered eating attitudes and behaviors

The 26-item Eating Attitudes Test (EAT-26), in its version validated for Brazilian girls (α = .82; Bighetti, Santos, Santos, & Ribeiro, 2004), assessed disordered eating attitudes and behaviors. The total score ranges from zero to 78 points, wherein the higher the score, the higher the risk of developing an eating disorder. Scores higher than 21 show a risky eating behavior (Garner, Olmsted, Bohr, & Garfinkel, 1982). The evaluation of the internal consistency of this scale for the present study sample was .79 at pretest and .71 at posttest.

##### Eating disorders symptoms

The Eating Disorder Diagnostic Scale (EDDS; Stice, Fisher, & Martinez, 2004) was used to evaluate symptoms of eating disorders. EDSS is composed by 23 items and the final score is calculated by the sum of the responses. Cronbach’s alpha value for the present study sample was .83 at pretest and .79 at posttest and its association with EAT-26 was .60 (*p* < .001).

#### Secondary outcomes

##### Depressive symptoms

The 20-item version of the Children Depression Inventory (CDI) was used to evaluate the presence and severity of depressive symptoms. The total score ranges from zero to 54 points, and the higher the score, the greater the presence of depressive symptoms. This scale has shown internal consistency (α = .81) and 1-factor structure among Brazilian adolescents between 7 and 17 years old (Wathier, Dell’Aglio, & Bandeira, 2008). The internal consistency of this scale for the present study sample was .90 at pretest and .71 at posttest.

##### Body appreciation

Body appreciation was evaluated through the 13-item Body Appreciation Scale (BAS). The final score was calculated by the sum of the responses and ranged from 13 to 65. For the BAS, the higher the total score, the higher the own body appreciation. The version used in the present study was the one translated into Portuguese and has shown internal consistency (α = .90; Caetano, 2011). Recently, the psychometric properties of this scale were provided for young Brazilian adolescents (Moreira, Lorenzato, Neufeld, & Almeida, 2018). The internal consistency of this scale for the present study sample was .90 at pretest and .91 at posttest.

##### Negative affect

The 15 items related to negative emotional states (e.g., sad, ashamed, angry, and nervous) of the Positive Affect and Negative Affect Scale (PANAS; Laurent et al., 1999) was used to evaluate the negative affect. The final score was the average of items responses. This scale has shown adequate internal consistency (.95 at pretest and .93 at posttest) and was significantly associated to the scores of CDI (*r* = .62; *p* < .001).

Both scales EDDS and PANAS are part of the Body Project Protocol. These scales are not validated for Brazilian adolescent girls. Thus, they were translated and back-translated and showed adequate internal consistency and significant correlations with measures of similar constructs, as described before. Since there are no validated measures to evaluate these outcomes, we included them in order to provide comparative results with others trials.

### Data analysis

Initially, a descriptive analysis of all variables was carried out, including mean and standard deviation values. Due to the distribution abnormality and sample size, non-parametric statistics were used. The preliminary analysis aimed to evaluate differences in the variables between the intervention group and the assessment-only controls at pretest, using the independent samples Mann-Whitney *U* test (attrition analyze).

In order to verify if the participants in the intervention condition had shown significantly greater reductions than the assessment-only participants, mixed repeated measures ANOVA was carried out for each outcome. We consider the allocation (intervention and assessment-only) as the between-subjects factor and the time (pre- and posttest) as the within-subjects factor. For the outcomes with significant group × time interactions, we made the post-hoc analysis, using the independent samples Mann-Whitney *U* test to verify differences between the groups in the posttest and the related samples Wilcoxon signed rank test to evaluate changes in the outcome from pre- to posttest. In addition, the effect size (Cohen’s *d*) was calculated and classified according to Cohen (1988): no effect (0–0.19), small (0.20–0.49), intermediate (0.50–0.79), large (0.80–1.29), and very large (> 1.30).

The analyses were performed in the IBM SPSS (Statistical Package for the Social Sciences) program for Windows, version 21.0, adopting the significance level of 95% (*p* < .05).

Moreover, the post-hoc tests for achieved power for each of outcomes were calculated, as well as for the average effect-size, using as parameters the observed effect-size and the final sample size. This analysis was performed using the GPower software, using a *p* = .05.

## Results

### Preliminary analysis

With regard to attendance, a total of 50,6% of intervention participants attended all four sessions (and completed the posttest assessment), 12.7% attended three sessions, 19% attended two sessions, and 17.7% attended one session. Among the assessment-only controls, the 35.5% completed the posttest assessment.

Descriptive statistics (means and standard deviations) for each outcome are presented in Table 2. Girls in the intervention group were classified as no dissatisfied (scores smaller than 80) and the assessment-only controls showed slight dissatisfaction (scores between 80 and 110) evaluated by the BSQ. Moreover, participants of both groups did not showed disordered eating attitudes and behaviors (scores smaller than 21), according to the EAT-26. There were no differences among participants in the two conditions in outcomes at pretest (see Table 2).

**Table 2:** Means and standard deviations for the intervention participants and assessment-only group on the outcome variables

Variables		Pretest	Posttest
		*M* (*SD*)	*M* (*SD*)
Body dissatisfaction (BSQ)	Body Project	77.49 (30.89)	55.81 (21.12)*
	Assessment-only	80.69 (36.49)	92.72 (43.52)^ϯ^
Sociocultural influences (SATAQ-3)	Body Project	75.76 (24.65)	59.11 (19.71)*
	Assessment-only	82.78 (23.58)	82.84 (27.96)^ϯ^
Eating attitudes (EAT-26)	Body Project	11.76 (9.02)	7.85 (6.76)**
	Assessment-only	14.33 (15.62)	17.47 (12.32)
Eating disorders symptoms (EDDS)	Body Project	10.32 (10.57)	7.03 (9.73)*
	Assessment-only	11.87 (11.87)	12.00 (10.64)
Depression symptoms (CDI)	Body Project	9.70 (7.97)	6.32 (4.67)*
	Assessment-only	11.86 (10.66)	13.44 (15.09)^ϯ^
Negative affect (PANAS)	Body Project	2.45 (1.26)	1.81 (0.66)*
	Assessment-only	2.23 (0.84)	2.34 (1.51)^ϯ^
Body appreciation (BAS)	Body Project	44.01 (10.56)	53.26 (8.98)*
	Assessment-only	45.73 (18.95)	41.40 (10.37)^ϯ^

*p < .001 and **p < .05 between pre- to posttest from related samples Wilcoxon signed rank test. ^ϯ^*p* < .05 between assessment-only and intervention from independent samples Mann-Whitney *U* test

### Comparison and differences between groups on pre-post scores in primary outcomes

There was a significant group × time interaction for pre- to post change for two of the primary outcomes (see Table 3): body dissatisfaction (BSQ: *F*[1,50] = 13.99. *p* < .001) and sociocultural influence of the media (SATAQ-3: *F*[1,49] = 14.40, *p* < .001). In both cases, the interaction reflected a greater pre- to post reduction in the BP group relative to the assessment-only controls, whose scores remained stable or increased from the pre- to posttest. The group × time interaction did not reach significance for eating attitudes and behaviors (EAT-26: *p* = .08) or eating disorders symptoms (EDDS: *p* = .06).

**Table 3:** Group × time interactions on the outcomes variables and effect sizes (Cohen’s *d*)

Variables	Group × time interaction^a^				
	*F*	*p*	*d*	Effect size^b^	Power^c^
Body dissatisfaction					
BSQ	14.00	.000	0.92	Large	0.99
Sociocultural influences					
SATAQ-3	14.40	.000	0.96	Large	1.00
Eating attitudes					
EAT-26	3.28	.076	0.46	Intermediate	0.94
Eating disorders symptoms					
EDDS	3.83	.056	0.56	Intermediate	0.99
Depressive symptoms					
CDI	8.24	.006	0.80	Large	0.99
Negative affect					
PANAS	6.64	.013	0.75	Intermediate	0.99
Body appreciation					
BAS	9.07	.004	0.71	Intermediate	0.99

^a^Data from Mixed ANOVA, with condition (assessment-only and BP) as between-subjects factor and time (pre and posttest) as within-subjects factor

^b^According to Cohen (1988)

^c^Calculating using the effect-size and the final sample size

### Comparison and differences between groups on pre-post scores in secondary outcomes

There was a significant group × time interaction for pre- to post change for all the secondary outcomes: depressive symptoms (CDI: *F*[1,48] = 8.24, *p* = .006), negative affect (PANAS: *F*[1,48] = 6.64, *p* = .013), and body appreciation (BAS: *F*[1,56] = 9.07, *p* = .004), with BP participants showing greater reductions on depressive symptoms and negative affect and greater increases on body appreciation than assessment-only participants.

The observed power for each of the outcomes evaluated, taking into account the final sample size (*n* = 62), are presented in Table 3. Considering the average effect size (*d* = 0.74), the power of the study was 0.99.

## Discussion

This trial evaluated the efficacy of a dissonance-based eating disorder prevention program among Latin-American adolescent females in Brazil. This study makes a novel contribution because it is important to determine whether interventions created in different cultural backgrounds are effective in different cultures. Moreover, this is the first trial conducted in Brazil to evaluate an eating disorder prevention program with a strong evidence-base, as have been shown by recent meta-analytic reviews (Le et al., 2017; Stice et al., 2007).

One explanation for why the BP is similarly effective in different cultural backgrounds, and among participants of this study, is that this intervention is participant-driven, what may make it naturally culturally adapting. For example, the Socratic questions used in the sessions allowed Brazilian girls to describe and criticize the body-ideal promoted in Brazil. Furthermore, research suggests that cultural pressure for thinness also appears to influence body image in young women in Brazil and that the prevalence of eating disorders is similar of which that has been noted in other countries (Fortes et al., 2013; Zordão et al., 2015).

The BP reduced body dissatisfaction, replicating effects from the large efficacy trial conducted in North America (Stice et al., 2006), as well as confirming the findings of recent meta-analytic reviews about the effects of dissonance-based intervention on body dissatisfaction (Le et al., 2017; Stice et al., 2007; Watson et al., 2016). Perez, Becker, and Ramirez (2010), using BSQ as measure of body dissatisfaction, also reported a reduction on levels of this outcome in young women of cognitive-dissonance intervention group. As body dissatisfaction is one of the most robust ED risk factors and significantly more common than clinical ED, interventions that are able to reduce body dissatisfaction should be encouraged and compose the public health efforts (Becker & Stice, 2017).

The BP also significantly reduced the influence of the media among the adolescent girls in the intervention group, relative to the assessment-only controls, which corresponded to a large effect (*d* = .96). This may be considered one of the main outcomes when evaluating the efficacy of this program since the reduction of the thin-ideal internalization, also accessed by the SATAQ-3, was the main mediator of the effects of this intervention (Stice, Marti et al., 2011). Several efficacy trials have found that BP produced greater reduction on thin-ideal internalization in adolescent girls and young women relative to assessment-only control conditions as well as relative to alternative intervention (Stice, Chase, Stormer, & Appel, 2001; Stice et al., 2000, 2006; Stice, Marti et al., 2008; Stice, Trost, & Chase, 2003). These results are confirmed in the present study.

The hypothesis that BP would reduce the ED attitudes and symptoms was partially supported. Although the effects for eating disorder attitudes and behaviors (EAT-26) and eating disorder symptoms (EDDS) did not reach significance, the effect sizes were medium (*d* = .46 and .56 respectively) and the power for this outcome was high (0.99). Also, the effect size for eating disorder symptoms is similar to or larger than the effect size found in trials (e.g., Stice et al., 2006; Stice, Butryn, Rohde, Shaw, & Marti, 2013). A potential explanation for these limited effects is that participants in the intervention had already low mean disordered eating attitudes and symptoms at pretest. Divergent results were found by Becker et al. (2005), in the universal version of the BP. Using the EAT-26 as measure of disordered eating attitudes and behaviors, the results indicated the efficacy of the program on this outcome, with greater reductions of the scores in the intervention group when compared to controls. McMillan, Stice, and Rohde (2011) did observe significant reduction in ED symptoms at posttest, but this effect was not significant by 3-month follow-up. The authors argued that maybe the self-reported measure are not sensitive enough to optimally measure change in ED symptoms.

Additionally, the BP significantly reduced depressive symptoms and the negative affect among participants (*d* = .80 and .75 respectively). Stice, Rohde, Durant, Shaw, and Wade (2013), using a CDI-like measure to evaluate this outcome, observed reduction on depressive symptoms in the intervention group. In general, most trials have found that the BP reduces negative affect (Becker et al., 2010; Stice, Marti et al., 2008), though effect sizes are typically smaller for this outcome.

Further, the BP increased body appreciation, with girls who participated of the intervention having significant higher scores than those in the assessment-only condition (*d* = .71). This result replicates the effects observed in a trial from the UK (Halliwell et al., 2015), in which the body appreciation increased among the adolescent girls (14 and 15 years old) in the intervention group, with effects ranging from weak to intermediate (*d* = 0.51). This finding is important because few eating disorder prevention trials have measured positive body image (Halliwell et al., 2015; Jankowski et al., 2017). Thus, it provides evidence that dissonance-based programs, in addition to reducing pathological aspects, also promote positive attitudes toward one’s body.

The interpretation of effect sizes is especially useful when comparing to other effects in the literature (Lakens, 2013). In this sense, the average effect size for the group × time interaction was *d* = 0.74, which reflects a greater effect size than those observed in similar trials (Halliwell & Diedrichs, 2014; Stice et al., 2006). For instance, in the efficacy trial of the BP (Stice et al., 2006), the average effect size was *d* = 0.59.

It is important to highlight that most of the trials that have been developed to evaluate the efficacy of the BP are selective (directed to young women with body image concerns) and results from selective prevention trials may not generalize to other sampling frames, such as young girls and women without body image concerns (Stice, Marti, et al., 2011). However, the BP has also shown efficacy when implemented universally to young women who were not screened for body image concerns (Becker et al., 2005). Participants in this study were concerned with their body image. Indeed, adolescent girls are considered as a high-risk group for body dissatisfaction and have demonstrated a normative body dissatisfaction (Duarte, Ferreira, Trindade, & Pinto-Gouveia, 2016; Littleton, 2008), which justify preventive efforts to reduce this outcome.

Despite these results, some limitations should be highlighted. First, we had a large dropout rate, which can be explained by the voluntary nature of the participation. Despite this, post-hoc tests pointed to a power of 0.99, considering the final sample size (*n* = 62), indicating that this study is able to identify real effects. Second, the assessment-only control condition was not a rigorous comparison condition because it did not control for demand characteristics inherent to randomized trials. But this seemed reasonable because the BP has significantly outperformed five alternative interventions in past trials, producing larger reductions in the outcomes. Third, we did not collect demographic data. However, it is important to highlight that body dissatisfaction and sociocultural influences have been observed in different socioeconomic levels as well as diverse demographic characteristics in Brazil (e.g., Laus et al., 2012). Fourth, we did not collect follow-up data. We argue that the pre- to posttest effects have been consistently reported in the literature. Also, it is difficult to keep the voluntary adhesion in long-term follow-ups in the Brazilian context, since it is not permitted to reimburse participants for completing assessments. However, given that the BP has produced significant effects through 3-year follow-up in multiple trials mitigates this concern somewhat. Finally, we did not use a more conservative *p* value to reduce the odds of chance findings because we were worried about missing true effects due to our relatively small sample size. We believe that replication is the most critical test of whether effects are reliable and the literature suggests that the BP does produce reliable effects across trials. A review of the literature indicates that 59 out of the 62 tests of the intervention effects for the core outcomes (thin-ideal internalization, body dissatisfaction, dieting, negative affect, and eating disorder symptoms) from pretest to posttest were significant in the 11 trials that Stice and his colleagues have conducted before the present trial (95%) which is reassuring because one would have expected only 3.1 out of these 62 effects to have emerged by chance (5%). Further, 34 out of the 47 tests of the intervention effects for the core outcomes were significant in 11 trials of the BP conducted by independent teams (72%), which is likewise much higher than the 2.4 out of 47 effects (5%) that would be expected based on chance. Thus, it seems highly unlikely that the effects reported herein are chance findings.

## Conclusions

In conclusion, the current findings support the usefulness of cognitive dissonance-based programs in the reduction of risk factors related to body dissatisfaction and to eating disorders and suggest that the participant-driven nature of the group discussions make it naturally culturally adapting. Advances such as the inclusion of measurements of body appreciation in the intervention are noteworthy. The novel evidence that the BP was efficacious for Latin-American adolescent females in Brazil generally extends evidence that this prevention program was similarly efficacious for various ethnic groups in North America and Europe (Halliwell et al., 2015; Rodriguez et al., 2008; Stice et al., 2014).

Results from the present study also provide directions for future research on the BP. It is fundamental to test the efficacy of the proposed program with a larger sample in Brazilian context and other unique cultures, using both the assessment-only control group and an active alternative comparison intervention and longer follow-ups. After confirming its efficacy, it is important to conduct studies aiming to evaluate the effects under real-world conditions, to confirm the effectiveness of this program. Last, it will be vital to evaluate how best to effectively implement this prevention program on a broad-scale basis, with the hope of reducing the incidence of eating disorders worldwide.

## Data Availability

The datasets used and/or analyzed during the current study are available from the corresponding author on reasonable request.

## Abbreviations

BAS: Body Appreciation Scale
BP: Body Project
BSQ: Body Shape Questionnaire
CDI: Children’s Depression Inventory
EAT-26: Eating Attitudes Test-26
ED: Eating disorders
EDDS: Eating Disorder Diagnostic Scale
PANAS: Positive Affect and Negative Affect Scale
SATAQ-3: Sociocultural Attitudes Toward Appearance Questionnaire-3
SPSS: Statistical Package Social Sciences software
